# Comparison of two behavioral tests for tinnitus assessment in mice

**DOI:** 10.3389/fnbeh.2022.995422

**Published:** 2022-10-10

**Authors:** Emily M. Fabrizio-Stover, Grace Nichols, Jamie Corcoran, Avni Jain, Alice L. Burghard, Christopher M. Lee, Douglas L. Oliver

**Affiliations:** Department of Neuroscience, University of Connecticut Health Center, Farmington, CT, United States

**Keywords:** gap-induced pre-pulse inhibition of acoustic startle (GPIAS), active avoidance (AA), inferior colliculus (IC), noise-induced hearing loss (NIHL), spontaneous activity

## Abstract

Animal research focused on chronic tinnitus associated with noise-induced hearing loss can be expensive and time-consuming as a result of the behavioral training required. Although there exist a number of behavioral tests for tinnitus; there have been few formal direct comparisons of these tests. Here, we evaluated animals in two different tinnitus assessment methods. CBA/CaJ mice were trained in an operant conditioning, active avoidance (AA) test, and a reflexive, gap-induced pre-pulse inhibition of acoustic startle (GPIAS) test, or both. Tinnitus was induced in awake mice by unilateral continuous sound exposure using a 2-kHz- or $\frac{1}{2}$ octave-wide noise centered at 16 kHz and presented at 113- or 116-dB SPL. Tinnitus was assessed 8 weeks after sound overexposure. Most mice had evidence of tinnitus behavior in at least one of the two behaviors. Of the mice evaluated in AA, over half (55%) had tinnitus positive behavior. In GPIAS, fewer animals (13%) were positive than were identified using the AA test. Few mice were positive in both tests (10%), and only one was positive for tinnitus behavior at the same spectral frequency in both tests. When the association between tinnitus behavior and spontaneous activity recorded in the inferior colliculus was compared, animals with tinnitus behavior in AA exhibited increased spontaneous activity, while those positive in GPIAS did not. Thus, it appears that operant conditioning tests, like AA, maybe more reliable and accurate tests for tinnitus than reflexive tests.

## Introduction

Subjective tinnitus, the perception of sound in the absence of an external stimulus, affects about 10% of American adults (Bhatt et al., 2016). Despite such a high prevalence, tinnitus research has been limited by the methods used to assess tinnitus in laboratory animals. Human patients can report the presence of tinnitus, while animals cannot. Animal models of tinnitus are crucial for developing new therapeutics for tinnitus, but these models must be validated with behavioral tests.

There are a variety of different behavioral tests for tinnitus and methods often vary across laboratories. Animal models of chronic tinnitus often use operant conditioning methods (Jastreboff et al., 1988; Bauer and Brozoski, 2001; Brozoski et al., 2002; Heffner and Harrington, 2002; Yang et al., 2011). In those methods, animals are trained to respond to sound stimuli, or the lack thereof, such that a change in the response indicates tinnitus (Kaltenbach, 2011). For example, animals may be trained to bar-press, lick, or climb only in response to sound, while being trained to suppress their responses in silence (Jones and May, 2017). “Tinnitus animals,” however, are presumed to no longer experience silence, so their behavior would be expected to be altered from non-tinnitus animals.

Active avoidance (AA) is one form of operant conditioning where animals are trained to avoid an aversive shock stimulus in response to sound stimuli. A reduction in percent avoidance in response to a particular sound may indicate tinnitus at that frequency. When multiple frequencies are used to evaluate the avoidance response; the frequency profile of the responses can give insights into the pitch of the tinnitus. Conditioned behaviors are useful because they can be used to determine not only the presence of a tinnitus percept but because they also can help to reveal the subjective qualities of the percept, such as pitch and loudness (Kaltenbach, 2011). However, conditioned behavioral tests are relatively time-consuming because training animals can require weeks before tinnitus induction.

Tinnitus can also be assessed with instinctual or reflexive tests. In a pre-pulse inhibition procedure, the acoustic startle response (ASR) is inhibited when a “warning sound” precedes the startle stimulus. A gap in a continuous background sound can also serve as a “warning stimulus” and inhibit the ASR. Turner et al. (2006) published a behavioral method, referred to as gap-induced pre-pulse inhibition of acoustic startle (GPIAS), that takes advantage of the ASR to assess tinnitus in animals. GPIAS uses narrow-band background noises of different frequencies to assess frequency-specific responses. If the animal has tinnitus, the percept is believed to “fill in the gap” and attenuate the ASR if the tinnitus and the background noise match in frequency. GPIAS has the advantage that animals do not need to undergo training, and, thus, less time devoted to testing is required as compared to that required when operant conditioning procedures are employed. However, the “filling in the gap” hypothesis has come into question. Recent studies have shown that human patients with tinnitus can still perceive gaps, suggesting that tinnitus does not affect gap perception (Campolo et al., 2013; Galazyuk and Hébert, 2015; Zeng et al., 2020).

Since the original publication of the GPIAS method (Turner et al., 2006), there has been little direct comparison of operant and reflexive behavioral tests in the same cohort of animals using a noise-induced hearing loss method of tinnitus induction. In the current study, the goal was to assess the same group of animals for tinnitus in GPIAS and AA to confirm that the results were consistent. After sound overexposure intended to induce tinnitus, the behavioral testing results were compared to determine if both tests indicated tinnitus in the same animals. Increased spontaneous activity has been correlated with behavioral evidence of tinnitus throughout the auditory pathway with operant conditioning methods (Kaltenbach et al., 2005; Bauer et al., 2008; Kaltenbach, 2011). At the level of the inferior colliculus (IC), it is well established that increased spontaneous activity is correlated with sound over-exposure (Mulders and Robertson, 2013; Wang et al., 2013; Gröschel et al., 2014; Ropp et al., 2014; Vogler et al., 2014); however, spontaneous activity in IC has not been correlated with tinnitus assessed with GPIAS (Berger et al., 2014; Coomber et al., 2014; Ropp et al., 2014; Longenecker and Galazyuk, 2016). So, we also compared the spontaneous activity in the IC between GPIAS and AA tinnitus-positive animals. We found that few mice exhibited evidence of tinnitus in both tests and increased spontaneous activity in the colliculus was found in mice with behavioral signs of tinnitus in active avoidance, but not if grouped by GPIAS results.

## Methods

### Animals

Experiments were performed on CBA/CaJ mice (Jackson Laboratories; Strain #000654, RRID:IMSR_JAX: 000654) of both sexes. All mice were purchased at the age of 4–8 weeks and then housed five in a cage employing a 12-h light/dark cycle with continuous access to food and water. Additional nesting materials were added as enrichment. All experiments were performed in accordance with institutional guidelines and the NIH Guide for the Care and Use of Laboratory Animals and were approved by the Animal Care and Use Committee at the University of Connecticut Health Center.

### Auditory brainstem response (ABR) and amplitude modulation following response (AMFR) recordings

Absolute thresholds were established before behavioral testing began *via* auditory brainstem responses (ABR) and amplitude modulation following responses (AMFR). Animals were anesthetized using a mixture of ketamine and xylazine (ketamine 10 mg/ml, xylazine 1.43 mg/ml) injected intraperitoneal (I.P.) or intramuscular (I.M.). Isoflurane (0.5%–2%) in 100% oxygen was used to maintain an anesthetized state as necessary. Animals were then placed on a gas anesthesia head holder (David Kopf Instruments, Tujunga, CA). Oxygen was provided *via* a nose cone at a flow rate of 0.5 L/min. Artificial tear ointment was applied. Body temperature was maintained at 36°C–37°C using a heating pad coupled to a rectal thermometer. Depth of anesthesia was assessed using the toe pinch reflex approximately every 30 min. Heart rate, respiratory rate, and O_2_ saturation were measured continuously *via* a pulse oximeter (MouseOx, Starr Life Science Corp, Oakmont, PA). Isotonic saline solution was administered subcutaneously, approximately every 30 min. Needle electrodes (Genuine Grass Reusable Subdermal Needle Electrodes, Natus, San Carlos, CA) were inserted under the skin under each ear and at the vertex of the head. If necessary, foam earplugs (CVS Health Foam Earplugs Advanced Protection, 30-decibel reduction rating, CVS Pharmacy, Woonsocket, RI) were used to help isolate responses from individual ears.

All recordings were performed in a sound-attenuated chamber (IAC, NY). Sounds were presented *via* a free-field speaker (Revelator R2904/7000-05 Tweeter, ScanSpeak, Videbaek, Denmark) at 15 cm from the head at midline and at an angle elevation of 45°. RMS sound levels of click trains having a 21 Hz repetition rate were calibrated at the position of the head within 5 dB from 3 to 70 kHz with a $\frac{1}{4}$-inch microphone (Precision Condenser Microphone, #377C01, PCB Piezotronics, Inc., Depew, NY). Amplitude modulated tones, narrowband noise, and wideband noise were calibrated similarly. ABR and AMFR recordings were made using an RZ6 Acoustic Processor and digitized at 25 kHz using a RA4L1 head stage (Tucker Davis Technologies, TDT, Alachua, FL). Using BioSig software (Tucker Davis Technologies), ABRs were evoked with 0.2 ms clicks with a presentation rate of 21 Hz at 0–90 dB RMS with alternating polarity and a step of 5 dB until the click ABR threshold was found. Responses to 512 click presentations were averaged and bandpass filtered (500–3,000 Hz). Hearing thresholds were determined by the level between the first detectable waveform and the last without a detectable waveform.

The full AMFR procedure is outlined in Burghard et al. (2019). Custom MATLAB code was used to generate and process the AMFR. The AMFR was evoked with a continuous 1/3 octave bandpass-filtered noise centered at 8, 11, 16, 22, or 32 kHz modulated by a 42.9 Hz sine wave, raised to the exponent 8. Stimulus presentation started at 30–40 dB above the click threshold and was decreased in 5 dB steps. The coherence of the responses over a range of modulation frequencies was measured, and coherence strength measured the extent to which the coherence at the modulation frequency differed from that at other nearby frequencies (see Burghard et al., 2019). If the coherence value was above 0.25 and the coherence strength was over 3, or if the coherence value was greater than 0.50 for five consecutive blocks (1 block = 8 epochs, 1 epoch = 10 cycles or minimum 250 ms), it was considered a “pass”. Five consecutive “passes” indicated that the stimulus was audible, and the intensity of the stimulus was decreased by 5 dB SPL. If 350 epochs were completed without five passes in a row, the stimulus was considered inaudible. The ABR and AMFR thresholds for each ear were collected with the opposite ear plugged with a foam earplug (CVS Health). Binaural recordings were made with no earplug inserted.

### Acoustic overexposure

Directly prior to sound overexposure, the right ear of the mouse was protected by a foam earplug to help preserve normal hearing in that ear. The earplugs were cut by hand to allow for a tight fit in the ear canal and cut individually for each animal. The animal was lightly anesthetized with 4% isoflurane in oxygen in an induction chamber and then the earplug was compressed, inserted into the ear, and allowed to expand. The fit was checked to ensure that the earplug filled the ear canal, and a liquid bandage (CVS Health Liquid Bandage) was applied over the surface of the earplug and pinna to secure it. The mouse was allowed to recover until sound exposure, at least 20 min. Animals were monitored throughout sound exposure to ensure the earplug was not removed.

Continuous acoustic overexposure was administered to awake mice in an anechoic chamber (IAC Acoustics, Naperville, IL, 28’ × 19’ × 17’) using a pair of Eminence N151M 8Ω speakers (Eminence Speaker LLC, Mulberry Pike Eminence, KY) modified with a Ferrofluid Retrofit Kit (Ferro Tec #020618-L11, Bedford, NH) and mounted on an Eminence H290B horn. The two free-field speakers faced each other one meter above the floor and were 11.5 cm from the center of the mouse holding cage. During sound overexposure, two mice were housed separately in two compartments of a holding cage configured from a small, aluminum autoclave basket mounted on a photographic tripod. Animals were exposed to 16 kHz-centered narrowband noise with a bandwidth of either 2 kHz or $\frac{1}{2}$ octave for 1 h. The sound was presented at either 113- or 116-dB SPL. Mice were continuously observed with a webcam during sound exposure and exhibited no signs of discomfort or distress. After sound exposure was completed, the earplug was removed, and the mice were returned to their home cage.

To confirm that the earplug spared the right ear from trauma, bilateral or right ear hearing thresholds and left ear thresholds were reassessed with ABR and AMFR at 2–4 weeks post sound overexposure. Animals with binaural or unexposed ear click ABR thresholds above 65 dB SPL were excluded from further behavioral testing.

### Behavioral testing and training

**Figure 1:**
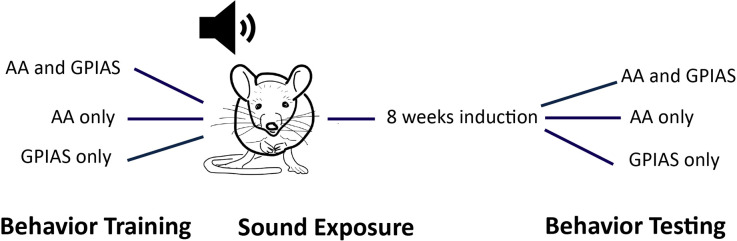
Behavioral training paradigm. Animals were trained either in active avoidance (AA) or gap-induced pre-pulse inhibition of acoustic startle (GPIAS) or both tests at once. Once training was finished, animals were sound exposed to generate tinnitus (113 or 116 dB 16 kHz 2 kHz wide noise band). Most were exposed with 113 dB as 116 dB caused an increase in dropped animals due to hearing loss (need number here). After 4–8 weeks to allow for tinnitus induction, mice were assessed in the tests that they were trained in before sound overexposure. Most animals that were trained in AA PRE were also trained in GPIAS post as the analysis did not require PRE data. However, it was impossible to test GPIAS PRE animals in AA post due to the need for training.

We used two behavioral assessments to assess tinnitus: gap-induced pre-pulse inhibition of acoustic startle (GPIAS) and active avoidance (AA). Performance on both tests was assessed before and two months after sound overexposure (Figure 1).

#### Gap-induced pre-pulse inhibition of acoustic startle (GPIAS)

The GPIAS protocol was adapted from Longenecker et al. (2018) and was performed using a startle audiometer system (Proxima Centauri Technologies, CA) designed by Michael Kinder. Animals were placed in a small cage on a pressure-sensitive plate inside a sound-attenuated chamber. Sounds were delivered with a free field speaker in the chamber, calibrated within 5 dB at 16 kHz with a $\frac{1}{4}$-inch microphone placed in the animal restraint (PCB Precision Condenser Microphone, #377C01, PCB Piezotronics, Depaw, NY). The amplitude (newtons) and time course (ms) of the acoustic startle reflex was measured with a load cell sensor calibrated with 100 g weights. Mice were allowed to acclimatize for 3 min prior to the test. The acoustic startle reflex was elicited with 20 ms, 95 dB SPL broadband noise bursts (broad noise from 3 to 30 kHz) in the presence of a background continuous narrowband noise (55 dB SPL, 1/3 octave bandwidth, center frequency of 11.3, 16, 22.3, or 32 kHz). Intertrial intervals varied randomly from 10 to 14 s with a one second step. Half of the trials consisted of background noise only with a startle stimulus presented at 120 ms (NO GAP). The other half of the trials had a 20 ms gap pre-pulse presented starting 80 ms before the startle stimulus (GAP). Mice were assessed at least five times, once or twice a week on non-consecutive days over multiple weeks post sound overexposure (Figure 1). Additionally, we assessed GPIAS performance in a subset of mice before sound exposure.

#### Active avoidance (AA)

The active avoidance method assesses tinnitus based on changes in response to a conditioned stimulus and was developed by Dr. Brad May (Johns Hopkins University; access is granted upon request from the authors^1^). In this method similar to lick avoidance (Jones and May, 2017), animals are trained to associate a tone as a warning of an adversive stimulus, in this case, foot shock. Silence is safe and no avoidance is necessary. The theory behind this is that mice with tinnitus will no longer experience silence. Rather, tinnitus will replace silence and become a “safe” sound. So, if a sound is played that is similar to the perceived tinnitus the animal will categorize the presented sound to the “safe” category and not the “warning sound” category, thus less likely to respond.

Mice were placed in a two-room shuttle box (PanLab, Harvard Apparatus, model LE916-918, Barcelona, Spain) connected by a gated port, housed in a larger sound-attenuated chamber. Load sensors placed underneath the two chambers of the shuttle box were used to track mouse positions. All sound stimuli were generated by a TDT RP2 processor. Tones of 32 frequencies were presented, centered at 8, 16.3, 22.3, and 32 kHz with a $\frac{1}{4}$ octave rove. Initially, tones were presented at 65–70 dB SPL, depending on the training success of the animal.

At the beginning of each session, there was a 5-min habituation period during which animals were free to move between rooms in the shuttle box. Mice then heard tones presented continuously for 15 s and had to move to the other room within 5 s after the onset of the tone. If the mice did not move, a shock was administered through the floor grid of the box using a Shock Generator with Scrambler (PanLab, LE100-26). If the mouse failed to avoid the shock initially but moved after shock onset, the shock and tone presentation stopped once it had crossed into the other chamber. If the mouse failed to relocate, the shock and tone presentation stopped after 15 s sound presentation (= 10 s shock duration). Inter-trial intervals varied randomly from 20 to 30 s. If the mouse relocated within the last 5 s before the end of the interval, a new intertrial interval began. Sessions lasted 45 min, thus each frequency was presented two-three times per session. If mice could perform the behavior, then the tones were presented at lower levels, with a minimum level of 60 dB SPL. Animals were trained to perform avoidance behavior with at least 75% accuracy across all frequencies for at least 5 days consecutively with stimuli presented at 60–70 dB SPL. Animals unable to meet that criterion were excluded from the study. Furthermore, if animals failed to improve their avoidance scores over 50% success after 4–5 days, they were excluded.

Eight weeks after sound overexposure, AA performance was assessed over five sessions, once or twice a week on non-consecutive days. Shocks were delivered on 50% of the trials to reduce the speed at which mice may re-learn to categorize sounds similar to their tinnitus percept as “warning sounds” and not as “safe” anymore.

Before sound overexposure, mice were trained in both assessments separately. That is, they would finish training in AA before being assessed in GPIAS, or assessed in GPIAS and then tested in AA. After sound overexposure animals were assessed in both tests in parallel during the same weeks, but not on the same days. So, a specific animal might be assessed in AA on Monday and GPIAS on Wednesday. Multiple training paradigms were used: trained in AA first and then GPIAS PRE sound exposure, trained in GPIAS first and then AA PRE sound exposure, trained in AA only PRE and then AA and GPIAS POST, trained in AA only PRE and POST, and trained in GPIAS only PRE and POST.

### Behavioral analysis

#### GPIAS

Startle amplitude was measured by taking the load cell sensor to the mouse’s weight and computing the RMS of the force during a 120 ms window following the onset of the startle stimulus. Viable startle trials were filtered based on the time course and amplitude of the startle force. First, trials in which the maximum force did not fall within 60 ms of the startle stimulus onset were removed. Then, the baseline amplitude of normal movement for each mouse was determined by averaging the maximum force for trials in the absence of an acoustic stimulus. Trials that were over one standard deviation baseline were included in the analysis. Trials needed to pass both the timing and amplitude requirements to be considered a valid startle response.

The tinnitus assessment was based on the ratio of startle amplitudes between GAP and NO GAP trials. Considering the evidence that sound overexposure can either increase or decrease the GAP: NO GAP ratio in CBA/CaJ mice (Longenecker et al., 2018), we assessed GPIAS performance using a modified ratio format. This strategy accounts for both increases and decreases in the GAP: NO GAP ratio and reduces variability between sessions by comparing the GAP: NO GAP and NO GAP: GAP ratio and taking the lowest (Longenecker et al., 2018). For each background frequency, startle amplitudes were separated into sets of 10 trials (5 GAP and 5 No GAP), resulting in nine sets. The lesser of the GAP/NO GAP or the NO GAP/GAP ratio was taken for each set, and all nine ratios were averaged.

The closer the modified ratio is to one, the less difference between GAP and NO GAP startle responses, consistent with the theory of tinnitus “filling in the gap” and attenuating the effect of the pre-pulse. When the background frequency matches or overlaps with the tinnitus pitch, the modified ratio at this frequency would be closer to 1, as if the mouse did not perceive the gap. We compared the modified ratio using an ANOVA with a *post-hoc* Tukey test over the five POST sound exposure sessions. A tinnitus frequency would be one with significantly higher (*p* ≤ 0.05) modified ratios.

#### AA

Performance on the AA task was recorded as correct or miss, based on whether the mouse moved between chambers before the onset of a shock or not. The percent correct avoidance responses were averaged from five days of post sound exposure testing. The tinnitus percept is hypothesized to interfere with AA performance when its pitch is similar to or overlapping with the presented stimulus. The mean correct avoidance was calculated across all 32 frequencies. The frequency with the worst avoidance score was compared to the mean performance using a one-sided student’s *t*-test. Significantly lower performance (*p* < 0.05) determined a positive tinnitus status.

#### Statistics

Statistical tests were done with OriginPro software (OriginLab Corporation, Northhamptom, MA). For GPIAS, data were analyzed with an ANOVA to determine differences between tested frequencies, followed by a Tukey *post-hoc* test. The Tukey test was selected as it adjusts for multiple comparisons but can also indicate what groups differ in our sample, and therefore what frequency was significantly different from the rest. AA data were analyzed with a Student’s *t*-test to compare the frequency with the worst performance to the overall mean performance to determine frequency-specific deficits.

### Multi-unit activity in the inferior colliculus

Recordings were performed in the same sound-attenuated chamber as were the hearing tests. Anesthesia was induced using ketamine/xylazine/acepromazine (90, 9, and 2.4 mg/kg body weight, respectively, IM or IP). Anesthesia was maintained using isoflurane in oxygen administered through a nose cone. The head was shaved, and 0.03 ml lidocaine hydrochloride (1%, subcutaneous) was administered at the incision site at the top of the head. The mouse’s head was then fixed in a position pitched forward 5 degrees from the horizontal stereotaxic plane in a stereotaxic frame with mandibular bars. An incision was made at the midline, and the skin and muscle were retraced. A craniotomy exposed both the right and left inferior colliculi. A stainless-steel screw (#0-80) was then inserted into the skull over the left cortex to serve as a reference electrode. A needle electrode was placed subcutaneously in the neck of the animal to serve as a ground. Following this, the dura mater covering the IC was removed.

Signals were collected with custom 32-channel, 2-shank linear silicon probes (length: 3 mm, 16 channels/shank, Neuronexus, Ann Arbor, MI). The shanks were spaced 400 μm apart, and the electrode sites were placed 100 μm apart. The probe was inserted with a manipulator (Scientifica, Uckfield, UK) at an angle of 10 degrees pitched caudal from the vertical. Electrode signals were digitized at 25 kHz with a TDT PZ5 amplifier and delivered to a TDT RZ5 processor.

All acoustic stimuli were generated with an RZ6 processor at a sampling rate of 200 kHz. Parameters of the acoustic stimuli were defined and digitally copied using user interface software “Synapse” and MATLAB function “SynapseLive” (TDT). Broadband noise bursts (3–50 kHz, 85 or 90 dB SPL, 100 ms duration, 2 Hz presentation rate) were played during electrode placement to confirm location within the central nucleus of the IC (ICC). Frequency response areas for each channel were obtained by presenting a sequence of pure tones and measuring the tone-driven response (200 ms duration, 4–64 kHz, 0–90 dB SPL, 10 dB, and 0.25 octave steps in a random presentation pattern). Each tone/sound level combination was presented five times. Spontaneous activity was collected over 1–2 min with no sound presentation.

## Results

We induced hearing loss in awake mice (*n* = 54) with a unilateral exposure to 113 or 116 dB SPL band-passed noise centered at 16 kHz, $\frac{1}{2}$ octave, or 2 kHz wide. Mice were evaluated with just AA, just GPIAS, or both behavioral assessments (Figure 1). Tinnitus assessment was performed eight weeks after sound overexposure.

### Categories of tinnitus results

**Figure 2:**
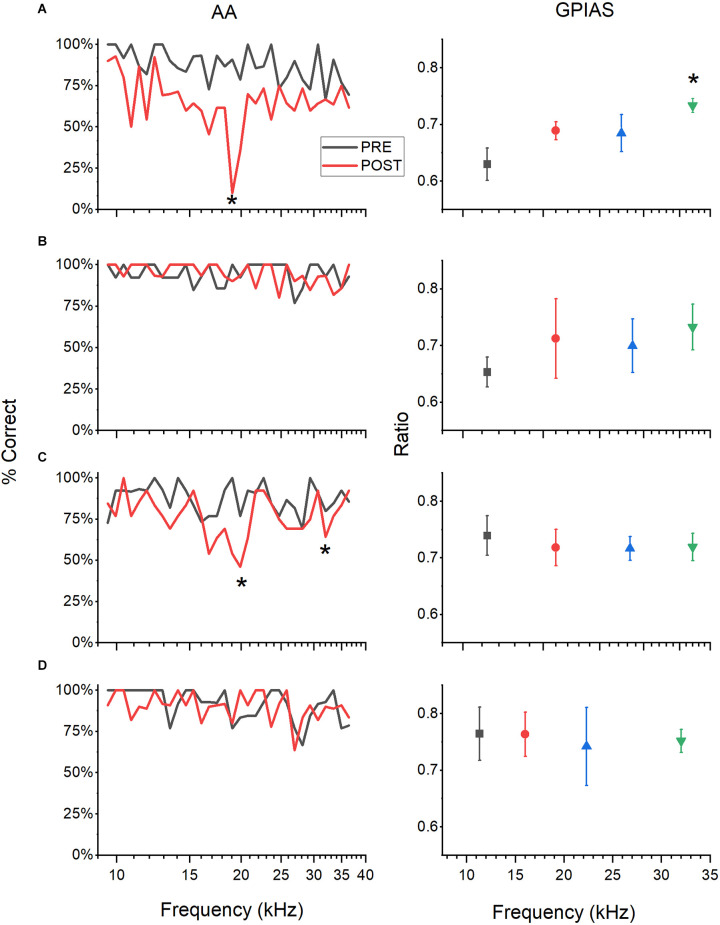
Examples of tinnitus behavior. GPIAS and AA results represented graphically for four example mice. AA results (left panels) show the percent successful avoidance averaged over five session POST sound exposure (red) and PRE (black) for comparison. GPIAS results (right panels) show the modified ratios (greater of GAP: NO GAP and NO GAP: GAP) of startle RMS at each of the four frequencies tested POST sound exposure over five testing sessions. Each row is one animal. **(A)** Animal has a significantly higher modified ratio at 32 kHz (One way ANOVA with Tukey test, *p* = 0.04). AA results show one frequency of deficit at 19.027 kHz (one sample *t*-test, *p* = 0.002). This animal is A+/G+. **(B)** Animal has no significant differences between frequencies for both GPIAS and AA testing. This animal is A−/G−. **(C)** Animal has behavioral evidence of tinnitus at 19.8 and 32 kHz in AA (*p* = 0.004 and *p* = 0.009 respectively, one sample *t*-test) but not in GPIAS. This animal is A+/G−. **(D)** Animal has behavior consistent with tinnitus at 16 kHz in GPIAS (one way ANOVA with Tukey test, *p* = 0.027), but no significant differences in AA. This animal is A−/G+. For the GPIAS data, only the POST results are shown, but for AA PRE and POST are shown. Statistics to determine the tinnitus frequency were done on POST data alone to help remove any effect that hearing loss may have on performance. *P < 0.05.

Animals assessed in both AA and GPIAS could exhibit tinnitus-positive behaviors in one task, both tasks, and neither task. Behavioral results were grouped into four categories: 1, positive in AA and GPIAS (A+/G+); 2, negative in both tests (A−/G−); 3, positive for tinnitus in GPIAS only (A−/G+), or 4, positive for tinnitus in AA only (A+/G−; Table 1). Figure 2 demonstrates the behavioral results from an example animal in each category. Figure 2A shows an animal with significant deficits in behavioral performance at specific frequencies in both the AA and GPIAS tests, although the deficits are at different frequencies. The AA results show one frequency of deficit (tinnitus frequency) at 19 kHz (one-sample *t*-test, *p* = 0.002). The mouse also had a significantly higher modified ratio in the GPIAS task at 32 kHz (One way ANOVA, *F*_(3,27)_ = 3.12, *p* = 0.044, Tukey test, *p* = 0.026). Animals with no tinnitus in either test (Figure 2B) had very flat frequency profiles and no significant differences in either AA and GPIAS testing frequencies. The mouse in 2C had behavioral evidence of tinnitus at 19.8 and 32 kHz in the AA task (*p* = 0.004 and *p* = 0.009 respectively, one-sample *t*-test) but not in GPIAS (one way ANOVA, *F*_(3,23)_ = 0.142, *p* = 0.933). In contrast, the mouse in 2D showed behavior consistent with tinnitus at 16 kHz in GPIAS (one-way ANOVA, *F*_(3,19)_ = 3.76, *p* = 0.032, Tukey test *p* = 0.027), but no significant differences in AA frequencies.

**Table 1:** Different sound overexposures have different distributions of tinnitus behavior.

**Type of Sound Overexposure**	**No.**	**A+/G−**	**A−/G+**	**A+/G+**	**A−/G−**	**Dropped (not included in n)**
113 dB 2 kHz wide	30	11 (52%)	2 (6%)	2 (9%)	8 (38%)	2
116 dB 2 kHz wide	8	2 (50%)	1 (25%)	0	5 (63%)	1
116+ dB $\frac{1}{2}$ octave	16	4 (40%)	6 (50%)	2 (29%)	7 (47%)	10

A+ is tinnitus positive in the active avoidance test. G+ is tinnitus positive in the GPIAS test. Three different sound exposure are shown. The n value and the total percentage for each category are shown. Percentages are of the total number of animals trained in that specific behavior paradigm, so they may not add up to 100%. For example, there were four A+/G− animals exposed to 116 dB 2 kHz wide, but two of them were positive in active avoidance behavior for all those tested in active avoidance. Animals exposed to 116 dB SPL trauma were more likely to be dropped due to excessive hearing loss, so most animals were exposed at 113 dB SPL. The percentage of A+ animals increased with 113 dB exposure, but the percentage of G+ animals decreased. These exposures were chosen to induce tinnitus (need citation here). The bandwidth of the sound was selected to create a more specific region of hearing loss. Therefore, the $\frac{1}{2}$ octave bandwidth exposure frequency was used to create a more specific loss. For example, the n of A−/G+ for 113 dB is 31, while the n for A+/G+ is 21. 113 has 21 tested in both and 10 tested in just GPIAS.

**Figure 3:**
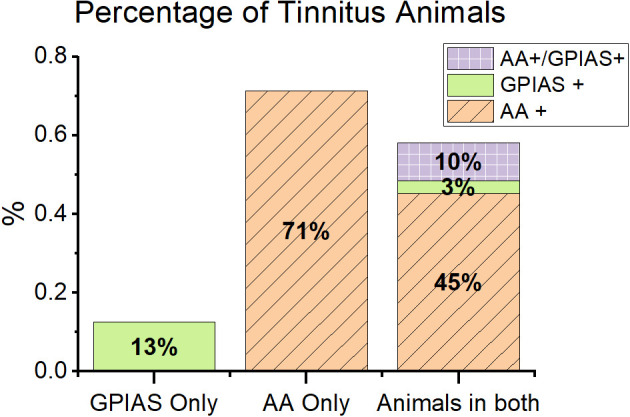
Animals showed different patterns of behavior in GPIAS and AA. Animals in the study are separated by training paradigm. GPIAS only = animals only trained in GPIAS (green). AA only = animals only trained in AA (orange with diagonal lines). Animals in both = all animals trained in both AA and GPIAS (purple with boxes). Overall, animals were more likely to be positive in AA than GPIAS. Very few animals were positive in both tests. Most of the animals positive in GPIAS were also positive in AA, very few were positive in GPIAS alone. Active avoidance training first increased the incidence of AA positive animals. N values: Only GPIAS *n* = 9, Only AA *n* = 7, AA with GPIAS post *n* = 28.

Overall, more animals exhibited behavioral evidence of tinnitus from AA testing (AA+) than from GPIAS testing. In the animals trained in just one behavior, 71% of AA only animals showed tinnitus behavior (AA only, *n* = 5 out of 7), while only 15% assessed in only GPIAS were tinnitus positive (GPIAS only, *n* = 3 out of 19, Figure 3). These proportions are similar for mice evaluated in both AA and GPIAS (AA + 57%, *n* = 16 out of 28, GPIAS + 14%, *n* = 4 out of 28). These results suggest that AA is more sensitive to potential tinnitus.

When assessed with both behavioral tasks, mice were typically positive for tinnitus behavior in only one task. The A+/G− group was the largest group with 13/28 mice (46.5%) testing positive for tinnitus, while the GPIAS+/AA− group had only one animal 3.5% (*n* = 1 out of 28). The AA−/GPIAS− had the second largest proportion of mice, with 39.2% of the animals being tinnitus negative in both tasks (*n* = 11 out of 28). Surprisingly, only a few mice demonstrated tinnitus positive behavior in both tasks (A+/G+, *n* = 3, Figure 3). Two of these mice did not have the same tinnitus frequencies across both tests, while one mouse had the same tinnitus frequency in both tests. Tinnitus is heterogenous, and the mismatch between frequencies in AA+/GPIAS+ mice and the low percentage of AA+/GPIAS+ mice suggests that AA and GPIAS may not identify tinnitus animals on the same basis.

Of the sound-exposed animals, 42.5% were male and 57.4% were female (*n* = 23 and 31, respectively). For all animals trained in active avoidance, including those just trained in active avoidance and those trained in both tests, males had a higher tinnitus positivity rate than females (70%, *n* = 7 out of 10, compared to 48%, *n* = 12 out of 25). For all animals trained in GPIAS, males also had a higher rate of tinnitus positive behavior than females (23.8%, *n* = 5 out of 21, compared to 3.8%, *n* = 1 out of 26).

**Figure 4:**
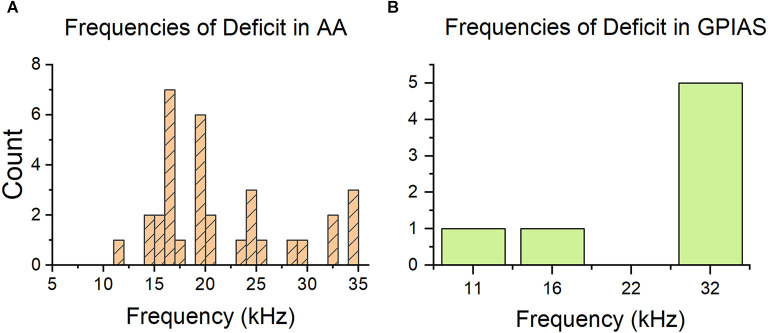
Distribution of tinnitus frequencies is different between behavior tests. **(A)** Histogram of frequencies indicated as tinnitus frequencies by the AA test. The count is calculated across all training paradigms. Most of the frequencies are at or above 16 kHz, which is the exposure frequency. **(B)** Histogram of frequencies of deficit indicated by the GPIAS test. Most frequencies are at 32 kHz, which is not consistent with the AA graph. GPIAS *n* = 7 frequencies and *n* = 7 mice, AA *n* = 33 frequencies and *n* = 21 animals.

With a sound exposure centered at 16 kHz, we would expect noise-induced damage in the cochlea to occur at about a $\frac{1}{2}$ octave above 16 kHz (or 22.6 kHz; Cody and Johnstone, 1981). Figure 4 shows the distributions of tinnitus frequencies for AA and GPIAS performance. While the AA+ frequencies are often at or above 16 kHz (average = 21 kHz, SD = 6.6 kHz), the majority of the GPIAS frequencies are at 32 kHz (average = 26.7 kHz, SD = 9.1 kHz). Furthermore, in GPIAS, no evidence of tinnitus was found at 22 kHz, where the maximum cochlear damage was expected. This discrepancy may be due to AA allowing for more potential tinnitus frequencies to be screened compared to GPIAS. GPIAS performance also may reflect the effect of high-frequency hearing loss and subsequently the reduced perception of a gap in noise.

### Effect of different sound overexposures

We investigated whether the type of sound overexposure affected the behavioral phenotypes of our tinnitus animals. Different sound exposure paradigms have been used for noise-induced tinnitus (Galazyuk and Hébert, 2015), so it was essential to compare the outcomes of the different acoustic trauma stimuli used for this study. Three narrowband noise sound exposure paradigms were used: $\frac{1}{2}$ octave wide at 116 dB SPL, 2 kHz wide at 116 dB SPL, and 2 kHz wide at 113 dB SPL, all centered at 16 kHz (Table 1). Sound exposure paradigms using 116 dB SPL frequently resulted in severe hearing loss that would interfere with behavioral performance (Table 1). Consequently, we adopted a 113 dB, 2 kHz-wide, 16 kHz centered sound exposure that resulted in a milder hearing loss phenotype.

For each sound trauma protocol, not all mice showed behavioral evidence of tinnitus (Table 1). When looking at all sound-exposed animals, including mice trained in two assessments and mice just trained in one, we found that 116 dB SPL exposures were more likely than the 113 dB SPL exposure to produce tinnitus positive behavior in GPIAS. The 113 dB SPL exposure was slightly more likely to produce tinnitus positive behavior in AA. Of the mice assessed with GPIAS and exposed at 113 dB SPL, only 6.6% were GPIAS+ (*n* = 2 out of 30); but, amongst the mice assessed with GPIAS and exposed to 116 dB SPL traumatic noise, 29.4% were GPIAS+ (*n* = 5 out of 17). In contrast, the percentages of AA+ mice did not differ greatly with different sound trauma paradigms. Of the mice assessed with AA, 65% (*n* = 13/20) were AA+ after 113 dB SPL exposure and 53.3% (*n* = 8/15) were AA+ after 116 dB SPL exposure.

**Figure 5:**
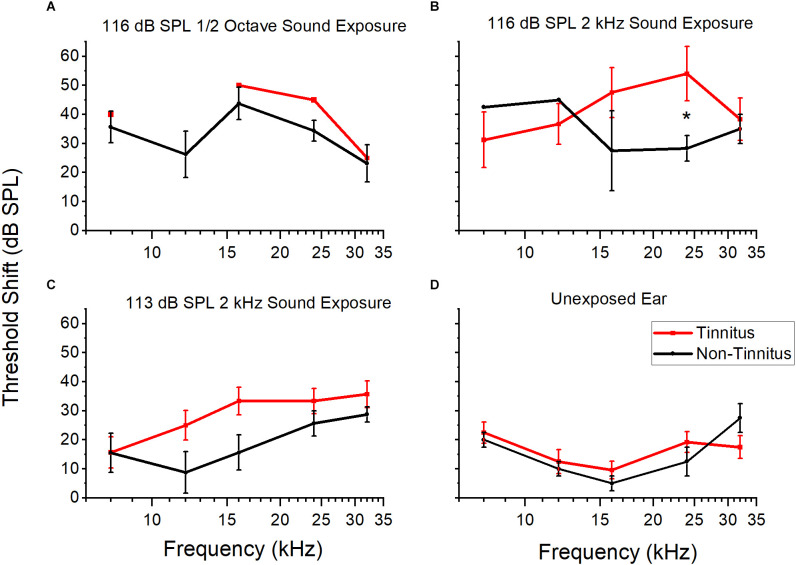
Threshold shifts of tinnitus and non-tinnitus groups after different sound overexposures. The mean and standard error for threshold shifts at five frequencies (kHz) approximately 2 weeks POST sound exposed for three different sound overexposure paradigms for tinnitus and non-tinnitus animals. **(A)** Threshold shifts after 116 dB SPL $\frac{1}{2}$ octave sound exposure in the exposed ear. Tinnitus *n* = 1, non-tinnitus *n* = 4. **(B)** Threshold shifts after 116 dB SPL 2 kHz sound exposure in the exposed ear. Significance at 24 kHz (*p* = 0.04, students two sample *t*-test). Tinnitus *n* = 6, non-tinnitus *n* = 3. **(C)** Threshold shifts after 113 dB SPL 2 kHz sound exposure in the exposed ear. No significant difference. Tinnitus *n* = 15, non-tinnitus = 8. **(D)** Threshold shift for unexposed, right ear across all sound exposures. No significant difference. Tinnitus *n* = 5, non-tinnitus *n* = 2. Tinnitus in red, non-tinnitus in black. Data shown as mean and standard error. The lack of standard error bars indicate only one data point for that frequency. *P < 0.05.

Different sound overexposure paradigms may result in different magnitudes of permanent threshold shift, which, in turn, could affect the likelihood of a tinnitus-positive diagnosis. Animals with more hearing loss may be more likely to have tinnitus behavior. To investigate a potential link between hearing loss and tinnitus, the hearing thresholds from AA+ and AA− mice were compared. Thresholds were determined using AMFR in the left and right ears (with contralateral ear plugged during recording) before and two weeks after sound exposure. Right ears had little hearing loss (Figure 5D). In left, exposed ears, tinnitus animals had a larger, but not significant, threshold shift across the entire audiogram compared to non-tinnitus animals (Figures 5A–C; 116 dB SPL $\frac{1}{2}$ octave wide could not be analyzed due to too low n value; 116 dB SPL 2 kHz wide *F*_(1,41)_ = 1.56, *p* = 0.16; 113 dB SPL 2 kHz wide *F*_(1,109)_ = 3.17, *p* = 0.07). However, at 24 kHz, the threshold shift was significant for the 116 dB SPL 2 kHz wide exposure (*p* = 0.04, student’s two-sample *t*-test). Coupled with preserved hearing in the unexposed ear, it seems unlikely that hearing loss was a confounding variable for behavioral performance (behavior was assessed with both ears open).

### Effect of sound overexposure on behavioral performance

**Figure 6:**
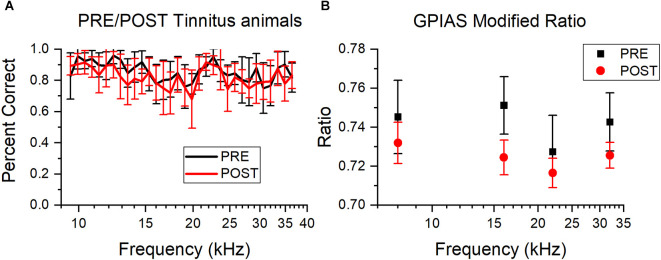
Sound overexposure does not affect AA or GPIAS performance: Comparison of percent correct trials for AA and GPIAS testing PRE and POST sound overexposure, plotted as mean and standard error. PRE is red, POST is black. **(A)** Average percent correct avoidance for non-tinnitus animals for all tested frequencies (kHz) PRE and POST. There are no significant changes between PRE and POST, but there is a small shift downwards for POST testing. PRE *n* = 14, POST *n* = 13. **(B)** Average modified ratio for non-tinnitus animals across all tested frequencies. There are no significant differences PRE and POST, but POST has slightly smaller ratios. PRE *n* = 9, POST *n* = 20.

It is possible that sound trauma can affect AA and GPIAS performance independently of tinnitus induction or hearing loss. So, performance in non-tinnitus animals before and after sound overexposure was compared for AA and GPIAS. There was a decrease in the percentage of correct avoidance trials in the AA non-tinnitus mice after sound trauma (Figure 6A), although this difference was not significant. Animals evaluated in GPIAS also had a slight, non-significant decrease in the modified ratio (One way ANOVA, *F*_(1,115)_ = 0.82, *p* = 0.06; Figure 6B). Although overall performance did not change significantly following sound overexposure, the GPIAS analysis is underpowered (alpha = 0.05, sample size = 116, power = 0.52) and we cannot rule out that there may be a change resulting from sound exposure. This can be ruled out for the results of the AA mice since their power was sufficient (alpha = 0.05, sample size = 575, power = 0.99). Therefore, in both assessments, we determined tinnitus status based only on post sound exposure performance, rather than PRE/POST-exposure performance changes.

### Spontaneous activity and tinnitus

**Figure 7:**
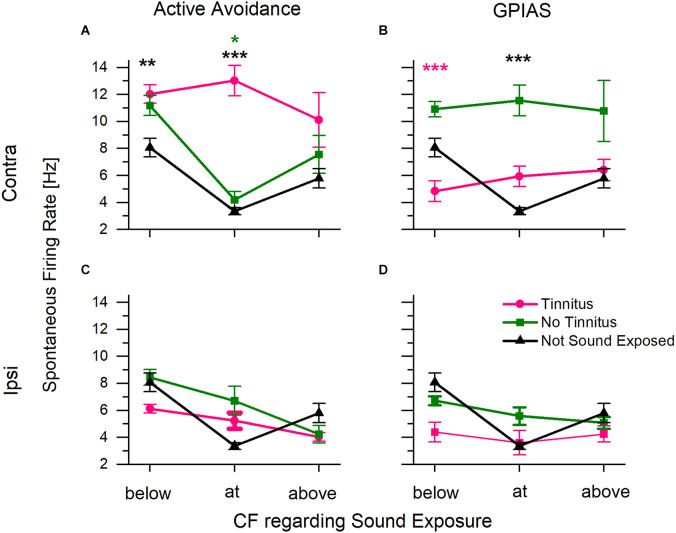
Average spontaneous firing rate in inferior colliculus collected from multi-channel electrodes. The characteristic frequency (CF) of each channel was determined using the collected frequency response area. Sound exposure frequency was 16 kHz. Data were plotted with the CF regarding sound overexposure. **(A)** Spontaneous firing rate from the IC contralateral to the sound exposed ear for animals assessed in AA. Includes animals trained in both tests. **(B)** Spontaneous firing rate from the IC contra to the sound exposed ear for animals assessed in GPIAS. Includes animals trained in both tests. **(C,D)** Spontaneous activity from the IC ipsilateral to the exposed ear for animals assessed in AA and GPIAS respectively. Tinnitus positive animals in pink, tinnitus negative animals in green, and control, unexposed animals in black. *P < 0.05, **P < 0.0005, ***P < 0.00005. A Pink * indicates significance from tinnitus, green indicates significance from no tinnitus, and black indicates significance to not sound exposed. AA+ *n* = 23 (18 trained in both tests, five trained in just AA), AA− *n* = 17 (13 trained in both tests, four trained in just AA), GPIAS+ *n* = 8 (four trained in both tests, four trained in GPIAS only), GPIAS− *n* = 28 (15 trained in both tests, 13 trained in just GPIAS), Control *n* = 3. N value is the number of animals.

Tinnitus behavior is associated with increased cellular excitability found throughout the auditory system, including spontaneous activity (Brozoski et al., 2002; Ma et al., 2006; Ropp et al., 2014). Therefore, multi-channel electrodes were used to compare the spontaneous activity of both ICs of our tinnitus-positive, tinnitus-negative, and control, unexposed mice. Sound overexposed mice had one ear plugged during sound overexposure, which allowed for recording from the IC contralateral to the exposed ear and a comparison to the IC ipsilateral to the exposed ear (Figure 7). The characteristic frequency (CF) of neurons at each electrode was determined based on the frequency response area. Since most mice were tested with both AA and GPIAS, we sorted them first according to their AA results into AA+ and AA−. We then resorted them according to their GPIAS results. Mice that were tested with AA only or GPIAS only were included in their respective groupings.

Spontaneous spike rates were significantly higher in the contralateral IC of the AA+ mice compared to the control unexposed mice. When the CF was at or below the sound exposure frequency, AA+ animals had higher spontaneous activity than control mice (Figure 7A; two-way ANOVA showed a statistically significant interaction between frequency (above/below/at CF) *F*_(2,3844)_ = 8.77, *p* = 0.03, and a main effect of tinnitus category *F*_(2,3844)_ = 26.2, *p* = 4.6*10^−12^, Scheffe *post-hoc*, below: *p* = 0.00005, at: *p* = 0.00001). The no tinnitus and control mice had similar spontaneous firing rates (Scheffe *post-hoc* tests, below *p* = 0.861, at *p* = 0.999, above *p* = 1).

In contrast, when the same mice were sorted according to GPIAS status, the GPIAS− mice had significantly higher rates of spontaneous activity than GPIAS+ mice at CFs below the sound exposure frequency (Figure 7B; two-way ANOVA showed a main effect for tinnitus category *F*_(2,3655)_ = 19.81, *p* = 0, but did not show a significant main effect for frequency *F*_(2,365)_ = 0.615, *p* = 0.540. Scheffe *post-hoc* test below *p* = 0.00061, pink asterisk). Furthermore, GPIAS- mice had significantly higher spontaneous rates at the sound exposure frequency when compared to control animals (Scheffe *post-hoc* test at frequency *p* = 0.00019, black asterisk). There were no significant differences between the control and GPIAS+ mice (Scheffe *post-hoc*, below *p* = 0.435, at *p* = 0.987, above *p* = 1). Thus, the GPIAS- mice were comparable to the AA+ mice with the highest spontaneous activity in IC.

Differences in spontaneous activity were not present in the IC ipsilateral to the sound trauma-exposed ear (Figures 7C,D). There were no tinnitus-specific differences in either AA+ or GPIAS+ animals. When taken together with exposed ear-specific differences in hearing threshold shifts (Figure 5), these data suggest that our earplug protocol effectively preserved hearing in one ear and limited the changes associated with sound trauma exposure and tinnitus in the ipsilateral IC.

## Discussion

In this study, we directly compared active avoidance and gap-induced pre-pulse inhibition of acoustic startle to assess an acoustic trauma-induced model of mouse tinnitus. When the same mice were assessed with AA and GPIAS, the behavioral results were often contradictory. Overall, more mice evaluated with AA exhibited behavioral signs of tinnitus, but very few mice showed tinnitus behavior in both assessments. When louder sound trauma conditions were used, mice evaluated with GPIAS were slightly more likely to exhibit tinnitus positive behavior, but the incidence of tinnitus was not correlated with the amount of hearing loss in the exposed ear. Sound overexposure did not appear to alter AA or GPIAS performance except at specific frequencies thought to represent tinnitus. Because the AA results often did not match the GPIAS results, it is not clear which of the two tests is a better assessment for tinnitus without a “ground truth” for tinnitus. While a definitive ground truth for tinnitus is lacking in mice, several lines of evidence associate increased spontaneous activity in the auditory pathway with tinnitus (Brozoski et al., 2002; Kaltenbach et al., 2004; Ma et al., 2006; Coomber et al., 2014; Kalappa et al., 2014; Ropp et al., 2014; Wu et al., 2016). We found that increased spontaneous activity in the IC contralateral to the exposed ear is associated with behavioral deficits in AA tinnitus performance but not GPIAS tinnitus performance. These results suggest that AA may be more sensitive and accurate in identifying tinnitus than GPIAS.

### Problems or sources of artifact

Noise-induced hearing loss models of tinnitus often use different types of acoustic stimuli to generate hearing loss. There is a fine line to walk between enough hearing loss needed to induce tinnitus (Jastreboff, 1990; Nondahl et al., 2011) and not too much loss so that animals can no longer perform behavioral tasks. To overcome this dilemma, one ear was plugged during sound overexposure to protect its hearing, and that allowed the animal to perform AA and GPIAS tests with relatively spared hearing (Figure 5). Turner and Larsen (2016) found that rats exposed to more intense noise had higher rates of hyperacusis, while those exposed to lower intensity noise had higher rates of tinnitus. We used two levels of sound exposures (116 dB SPL and 113 dB SPL) to induce tinnitus. The 116 dB SPL sound exposure resulted in higher threshold shifts, more dropped animals, and more GPIAS+ animals than the 113 dB SPL exposure (Table 1). However, the 113 dB SPL sound exposure resulted in more AA+ animals and fewer animals dropped due to hearing loss, consistent with lower sound exposures resulting in more tinnitus positive behavior.

Our different sound overexposure paradigms may have produced different magnitudes of permanent threshold shift, which, in turn, changes the likelihood of a tinnitus-positive diagnosis. We compared the average post sound exposure threshold shifts between tinnitus and non-tinnitus animals and found no significant differences overall (Figures 5A–C). Furthermore, there was very little threshold shift in the unexposed, protected ear (Figure 5D). Therefore, it is unlikely that noise damage in the exposed ear was a confounding variable for AA or GPIAS performance.

Our sound overexposure was performed in an anechoic chamber on unanesthetized mice. Acoustics in a closed field, such as an ear tube, can be harder to control and high frequencies can be blocked easily, changing the spectrum of the traumatic noise. Likewise, sound exposure in a reverberant environment may also suffer from the presence of standing waves that alter the spectral composition of the sound. Sound exposure in an anechoic chamber allowed for excellent control over the acoustics and a uniform sound environment (Mwilambwe-Tshilobo et al., 2015; Jones and May, 2017). The use of anesthesia is another factor that may influence tinnitus induction since any anesthetic agent that raises the threshold of hearing could reduce the damage induced by sound overexposure. It is common to induce tinnitus by exposing anesthetized mice to a loud sound (Longenecker and Galazyuk, 2011; Wu et al., 2016; Sturm et al., 2017). When compared to unanesthetized mice, mice anesthetized with pentobarbital, isoflurane, or halothane anesthesia have less severe auditory threshold shifts after noise trauma (Chung et al., 2007), suggesting a protective effect. It is unclear how anesthesia influences the development of chronic tinnitus, but isoflurane has been shown to acutely diminish the amplitude of temporary tinnitus (Norman et al., 2012). Therefore, it is possible that awake and anesthetized sound overexposures could result in different patterns of auditory trauma and different (behavioral) phenotypes of tinnitus. The extent to which our open-field, unanesthetized sound overexposure paradigm contributes to our behavioral phenotype of tinnitus is unknown.

One potential pitfall with GPIAS is that there are different sources of variability that can influence the results, variability between GPIAS protocols, and variability intrinsic to the ASR response. To illustrate the inconsistency of GPIAS methodologies, Galazyuk and Hébert (2015) outlined seven mouse studies that used seven different types of protocols to test GPIAS. For ASR variability, Longenecker et al. (2018) outline variables that can influence the ASR, including inter-trial variation, circadian rhythm, sex differences, weight, sensory adaption, and the incidence of both gap-induced facilitation and inhibition. Analytical methods have been adopted to address and reduce the variability of GPIAS performance (Longenecker et al., 2018). We interpreted the GPIAS results based on the modified ratio of RMS amplitudes because it has been shown to limit the variability both within and between GPIAS sessions. Furthermore, CBA/CaJ mice demonstrate both gap-induced facilitation and inhibition, and the modified ratio takes this into account.

One problem with active avoidance is that it is a behavioral test that relies on negative reinforcement for training a behavior. Negative reinforcement can stress animals, which can, in turn, affect behavioral performance. To help mitigate stress, we provided all our mice, regardless of training paradigm, with secondary enrichment in their home cages and monitored their learning in AA. If an animal did not reach criterion performance (75% correct across all frequencies) within five training sessions or its percent correct performance decreased over successive training sessions, we dropped the animal from the study.

Because AA is an operant behavior, the sound trauma exposure followed by an eight-week tinnitus induction period creates a scenario where the animal may forget the conditioned avoidance response. Almost all the mice in this study were able to perform the AA tests at a similar level to before sound overexposure (for non-tinnitus frequencies), but the loss of the learned response is a potential issue with AA testing. In addition, AA requires a greater time commitment than GPIAS since mice need to train before sound exposure and then be tested again after sound exposure.

On the other hand, AA performance may be less variable than GPIAS performance because it is measured as a discrete go/no go response, while GPIAS performance is measured as a continuous data ratio. AA also has advantages over other operant conditioning tests. First, it does not require food or water deprivation, which can cause chronic physiological stress that affects behavior (Faraco et al., 2014). Second, AA uses negative reinforcement, which allows for faster training than positive reinforcement (LeDoux, 2000) and mitigates the time investment needed to train animals. Foot-shock exposure can lead to stress, but avoidable foot-shock does not raise corticosteroid levels above those of animals exposed to the same environment but with no foot-shock (Van der Borght et al., 2005; Lesburguères et al., 2016).

### Previous comparison of operant and reflexive tinnitus assessment

We found that AA and GPIAS yielded often contradictory results. In the only other direct comparison of which we are aware, Turner et al. (2006) reported that their GPIAS results were highly consistent with an operant gap detection test for tinnitus conducted in the same animals. This discrepancy may reflect differences in the model species, as well as the behavioral and tinnitus induction methods. Turner et al. (2006) studied rats, while the present study used mice. Both operant methods were go/no-go tasks, but in AA the mice had to initiate an avoidance behavior when any tone was played, while in the operant gap detection the rats had to stop bar pressing to any tone. After a unilateral sound overexposure to a 116 dB, one octave-wide noise centered at 16 kHz under anesthesia, rats were found to have chronic tinnitus at 10 kHz (Bauer et al., 1999; Bauer and Brozoski, 2001). In contrast, our mice were overexposed to narrower-band stimuli centered at 16 kHz at 116 or 113 kHz dB while awake, but the tinnitus frequencies were routinely higher in frequency than the overexposure stimulus in both AA and GPIAS results. The studies also differ in the stimuli used for GPIAS testing. Our GPIAS method tested gap inhibition in four 1/3-octave noises covering the same frequency range as the AA method. In contrast, Turner et al. (2006) used gaps in broadband or 2 kHz-wide noises, but only tested two narrow band noises centered at 10 kHz or 16 kHz. They found there was less inhibition of bar-pressing at 10 kHz consistent with their operant gap detection results. It is possible that if a wider range of center-frequencies were tested, similar to the range of frequencies in the operant gap detection, the frequency identified with GPIAS as tinnitus would have been found at a frequency other than 10 kHz.

### Learning and behavior

Behavioral tests for tinnitus, such as AA and GPIAS, may not be accurate if animals learn to distinguish their tinnitus from the acoustic cue. Our AA testing sessions were conducted with only 50% shock reinforcement to delay the mice from learning to distinguish their tinnitus from the test stimulus. This learning was further delayed for both our AA and GPIAS testing since there were at least 2 days to a week between each behavioral testing session after sound overexposure.

Tinnitus testing with GPIAS hinges on the theory that animals cannot learn to distinguish between internal and external sound. The implication is that the tinnitus percept “fills the gap” and masks the perception of a gap in noise (Turner et al., 2006). However, human patients with tinnitus easily learn to distinguish their tinnitus from gaps in external sounds (Fournier and Hebert, 2013). Animals with tinnitus may do the same. It is possible that during gap trials of GPIAS tests, mice learn to distinguish the gaps in the external sounds from their internally generated tinnitus, and this may result in less startle response and more variability.

The results of our AA paradigm could be explained by two potential mechanisms. One possible mechanism is that the tinnitus “masks” the warning sound if the tinnitus is similar to the frequency of the warning sound. A second possibility is that, in active avoidance, the tinnitus percept is easily distinguished from external sound. During initial training, mice learn that silence is safe and that when a tone is presented, they need to move to avoid a shock. After sound exposure, the tinnitus mouse no longer hears silence, and the tinnitus percept is generalized to become a “safe” sound. A cue presented close to the tinnitus frequency would also be categorized as “safe” and the mouse is less likely to avoid the shock. Jones and May (2017) discuss this possibility when developing a lick-suppression protocol where the tinnitus frequency becomes a cue for safe drinking. A “safe” sound test is advantageous because it may be more resilient to tinnitus percept discrimination (Jones and May, 2017).

### Attention and tinnitus

In human patients, attention may play a role in triggering tinnitus and in the management of tinnitus. A top-down modulation of subcortical structures may contribute to the perception of tinnitus (Roberts et al., 2013). Attention has also been shown to play a role in auditory perception and tinnitus in animals. Tinnitus rats, when compared to non-tinnitus and control rats, showed more vigilance to unpredictable sounds, suggesting an increased role of attention in behavioral assessments for tinnitus (Brozoski et al., 2019).

There is evidence that pre-pulse inhibition of the ASR can be affected by top-down modulation, including attentional modulation. In rats, pre-pulse inhibition can be enhanced when the pre-pulse is coupled with a shock (Li et al., 2008; Du et al., 2009). However, our GPIAS test does not involve a noxious stimulus for the mouse. Intertrial intervals were randomized for AA and GPIAS so that the stimulus onset would be unexpected, but negative reinforcement is only used in AA testing. For this reason, mice may be more likely to be alert and attentive to their surroundings in AA. Conversely, GPIAS has no negative consequence for the mice if they do not startle. Therefore, AA may require more attention from animals and affect how they perceive their tinnitus.

### Spontaneous activity

Increased neuronal excitability across multiple auditory nuclei, including the IC, commonly occurs in animal models of tinnitus (Brozoski et al., 2002, 2007; Kaltenbach et al., 2004; Ma et al., 2006; Bauer et al., 2008). The IC has been shown to be important for the generation of tinnitus in both noise-induced and drug-induced models of tinnitus (Chen and Jastreboff, 1995; Henry et al., 2014; Ropp et al., 2014; Vogler et al., 2014; Smit et al., 2016). In the present study, we examined spontaneous activity on both sides of the IC. We saw a lateralized increase in spontaneous activity in the IC contralateral to the sound trauma exposed ear in mice positive for tinnitus in AA but no increase in the IC ipsilateral to the exposed ear.

Increased spontaneous firing in tinnitus animals following tinnitus-induction has been shown using multiple behavioral models of tinnitus. Tinnitus animals of multiple species identified with operant conditioning methods showed increased spontaneous activity in the auditory system [Chinchillas (Brozoski et al., 2002; Bauer et al., 2008), hamsters (Kaltenbach et al., 2004)]. Gap detection tests used to assess tinnitus behavior also showed increased activity in the auditory system. In a guinea pig study, the increased spontaneous firing rate was correlated with GPIAS tinnitus behavior (Wu et al., 2016). Rats with GPIAS tinnitus have increased rate level function slope in the medial geniculate body positively correlated with tinnitus score (Kalappa et al., 2014). However, the evidence of increased spontaneous activity in the IC specifically, as correlated with GPIAS tinnitus, is mixed. Coomber et al. (2014) in guinea pigs, found that all noise exposed animals had elevated spontaneous activity in the IC, regardless of GPIAS tinnitus status. Furthermore, gap detection thresholds in the IC were determined to be much shorter than the gap durations commonly used in GPIAS (Berger et al., 2014). Ropp et al. (2014) found that in unilaterally exposed rats, GPIAS tinnitus positive animals did not have differences in spontaneous activity between the exposed and unexposed ICs. Similarly, our results show that GPIAS positive animals do not have increased spontaneous activity in the IC, while the AA mice do have increased spontaneous activity in the IC opposite the exposed ear.

Some human patients perceive their tinnitus as localized to one ear (Al-Swiahb and Park, 2016). Lateralization of the tinnitus percept implies that the neurological changes resulting in tinnitus may be asymmetric. Evidence of lateralized tinnitus-dependent changes in the IC is mixed in animal models. Behavioral testing in rats showed that unilateral sound overexposure resulted in more false positive responses to silence on the side of the exposed ear, supporting the hypothesis that unilateral overexposure can result in lateralized tinnitus (Heffner, 2011). However, in unilaterally exposed rats not separated by tinnitus status, there was no difference in spontaneous activity between the contra- and ipsilateral ICs to the sound exposed ear (Ropp et al., 2014).

### Does tinnitus fill the gap in GPIAS?

The assumption underlying GPIAS is that tinnitus “fills in the gap” and reduces gap detection, leading to less inhibition of acoustic startle. However, recent studies have raised issues with GPIAS as an accurate test for tinnitus. Mice with acoustic trauma meant to induce tinnitus do not show deficits with GPIAS unless the gap was placed directly before the startle stimulus (Hickox and Liberman, 2014). Another study in rats shows that the behavioral threshold for gap detection does not change after a dose of sodium salicylate meant to induce tinnitus, suggesting that salicylate-induced tinnitus does not affect gap detection (Radziwon et al., 2015). So, changes in the prepulse inhibition of acoustic startle may not always be reliable indicators of tinnitus.

In human subjects, tinnitus does not interfere with auditory and speech perception (Zeng et al., 2020) or frequency-specific gap detection (Fournier and Hebert, 2013). This suggests that people with tinnitus can distinguish between external and internal sounds. Furthermore, Campolo et al. (2013) and Boyen et al. (2015) did not find gap detection deficits in human subjects at all. These studies suggest that gap-detection tests may be useful for assessing other auditory disorders such as hyperacusis, but not tinnitus. This is consistent with our results showing very few mice with tinnitus-positive behavior in both AA and GPIAS.

## Conclusion

This study attempts to clarify the confusion surrounding the benefits of different behavioral models for noise induced tinnitus in mice and emphasizes that not all tinnitus assessments may evaluate the same phenomena. We found AA to be a more precise and reliable test for tinnitus behavior in mice following noise-induced hearing loss. Mice with tinnitus behavior in AA showed a clear increase in spontaneous activity in the inferior colliculus. In contrast, the hypothesis underlying the GPIAS test for tinnitus has been called into question, and our GPIAS positive mice did not have increased spontaneous activity. Our results suggest that with our sound overexposure in awake mice, the behavioral phenotypes from the AA and GPIAS tests are driven by different auditory pathways and that tinnitus positive behavior in AA is correlated with electrophysiological evidence of tinnitus in the inferior colliculus.

## Data Availability Statement

The raw data supporting the conclusions of this article will be made available by the authors, without undue reservation.

## Ethics Statement

The animal study was reviewed and approved by Animal Care and Use Committee at the University of Connecticut Health Center.

## Author Contributions

EF-S: study design, data collection, analysis, and writing—first draft. CL: code writing and data collection. GN, JC, AJ, and AB: data collection and analysis. DO: study design, writing, and funding acquisition. All authors contributed to the article and approved the submitted version.

## Funding

This work was financially supported by the DOD, United States Army, MEDCOM, and Congressionally Directed Medical Research Programs (CDMRP)—W81XWH-18-1-0135.

## Conflict of Interest

The authors declare that the research was conducted in the absence of any commercial or financial relationships that could be construed as a potential conflict of interest.

## Publisher’s Note

All claims expressed in this article are solely those of the authors and do not necessarily represent those of their affiliated organizations, or those of the publisher, the editors and the reviewers. Any product that may be evaluated in this article, or claim that may be made by its manufacturer, is not guaranteed or endorsed by the publisher.

## Footnotes

^1^ https://activeavoidance.wordpress.com
